# Lower Airway Diseases in the Paediatric Population: A Two-Year, Single-Centre, Retrospective Study

**DOI:** 10.3390/jcm14020384

**Published:** 2025-01-09

**Authors:** Anna Ferrero, Antonia Versace, Marco Denina, Giulia Spagna, Alessandra Vincenza Fera, Margherita Conrieri, Claudia Bondone

**Affiliations:** 1 Paediatric Unit, Ospedale Regina Montis Regalis, 12084 Mondovì, Italy; anna.ferrero2@aslcn1.it; 2 Department of Pediatric Emergency, Regina Margherita Children’s Hospital, Città della Salute e della Scienza, 10126 Turin, Italy; aversace@cittadellasalute.to.it (A.V.); mconrieri@cittadellasalute.to.it (M.C.); cbondone@cittadellasalute.to.it (C.B.); 3 Paediatric Infectious Diseases Unit, Regina Margherita Children’s Hospital, University of Turin, Città della Salute e della Scienza, 10126 Turin, Italy; 4 Department of Paediatrics and Public Health, Regina Margherita Children’s Hospital, University of Turin, Città della Salute e della Scienza, 10126 Turin, Italy; giulia.spagna@unito.it (G.S.); alessandravincenza.fera@unito.it (A.V.F.)

**Keywords:** lower airway diseases, respiratory failure, bronchiolitis

## Abstract

**Background**: Lower airway diseases in children are one of the major causes of hospitalisation. This study aimed to evaluate the characteristics of children admitted to a tertiary pediatric hospital diagnosed with lower airway disease and to identify differences between age groups and the two years of the study. **Methods**: In this single-centre retrospective observational study, demographic and clinical information about children hospitalised in the emergency pediatric ward and diagnosed with lower respiratory disease from 1 June 2021 to 30 June 2023 were retrospectively reviewed. **Results**: A total of 410 episodes of hospitalisation for lower airway diseases were registered. In 83.9% of cases, the patient needed hospitalisation for respiratory failure, and children <1 year of age were at higher risk. Rhinovirus and respiratory syncytial virus (RSV) were the leading causes of lower respiratory tract infections. No death has been recorded. In 8.8% of cases, the patient was admitted to the Pediatric Intensive Care Unit. In 2021–2022, we recorded more hospitalisations for bronchiolitis with RSV as the primary pathogen detected and more patients were admitted to the hospital for respiratory failure. In 2022–2023, we registered more admissions for bacterial pneumonia and the need for intravenous therapy. **Conclusions**: Lower respiratory tract diseases are frequent in the pediatric population, and the risk of respiratory failure is higher. Analysing the differences between the two years of study, we underline how the COVID-19 pandemic has changed the epidemiology of acute respiratory infections in children.

## 1. Introduction

Lower airway diseases are one of the major causes of hospital admission for children. They include acute lower respiratory tract infections (LRTI) and their complications, recurrent wheezing, and asthma [1].

Acute respiratory infections are the most common cause of pediatric emergency department visits. Acute LRTI includes tracheitis, bronchitis, bronchiolitis, and bronchopneumonia.

Pneumonia and bronchiolitis are a leading cause of mortality and morbidity in young children worldwide. They are the sixth leading cause of mortality for all ages and the leading cause of death among children younger than 5 years [2].

Viral bronchiolitis is the most frequent lower respiratory tract infection in infants less than twelve months of age [3]. The most common causative agent is respiratory syncytial virus (RSV). RSV contributes substantially to the global burden of morbidity and mortality in children, especially during the first 6 months of life [4].

Pneumonia is common in all age groups and the etiology changes according to the age of the child and the setting in which the infection is acquired. Viral pathogens are a prominent cause of LRTI in infants older than one month and younger than five years of age. *S. pneumoniae* is the most common bacterial pathogen in children aged 3 weeks to 4 years of age, whereas Mycoplasma pneumoniae and Chlamydia pneumoniae are the most frequent bacterial pathogen in children older than 5 years of age [5,6].

Asthma is a chronic inflammatory condition of the lower airways; it is the most common chronic disease in children in resource-rich countries and it can be diagnosed in children older than 5 years. Recurrent wheezing in preschool children is also common, frequently triggered by viral respiratory tract infections, and usually resolves during the preschool years [7,8].

The clinical presentation of lower airway diseases includes cough, tachypnoea and respiratory distress. In LRTI, fever is often present. Most children with lower airway diseases are treated on an outpatient basis. Causes of hospitalisation include acute respiratory failure (it may be defined in terms of blood gas abnormalities: partial pressure of oxygen in the blood < 60 mmHg, partial pressure of carbon dioxide in the blood > 55 mmHg and saturation level of oxygen in hemoglobin (SaO_2_) < 90%) and the need for intravenous therapy.

Our study aimed to retrospectively describe the epidemiological characteristics, admission features, clinical management, and outcomes of patients hospitalised with a diagnosis of lower airway disease. We tried to identify different features between different age groups and between the two years of the study.

## 2. Materials and Methods

This single-centre retrospective observational study was conducted at Regina Margherita Children’s Hospital in Turin, Italy. The demographic and clinical information about children hospitalised and diagnosed with lower respiratory disease from 1 June 2021 to 30 June 2023 were retrospectively extracted from the electronic health records. We analysed hospitalisation episodes, including patients admitted to the emergency pediatric ward of our hospital, and the same patient admitted again was considered as a new case. We defined lower airway disease as one of the following conditions: bronchitis, bronchiolitis, bronchopneumonia, pneumonia or its complications, asthma, and recurrent wheezing. These diseases were diagnosed based on clinical, radiological and laboratory evidence. We excluded patients with a diagnosis of upper respiratory tract infection.

The medical record databases of our hospital were screened for information relative to the demographic profile of children (age, sex, and origin), symptoms at admission, reason for hospitalisation, outcome (length of stay, admission to pediatric intensive care unit—PICU, surgical procedures performed), and diagnosis at discharge. Microbiological information was gathered through the results of polymerase chain reaction (PCR) analysis of nasopharyngeal swabs, RSV antigenic nasopharyngeal swabs, serologies, bacterial cultures, and pneumococcal urinary antigens. We also recorded radiological findings from lung ultrasound, chest radiography and computed tomography (CT) scans of the lung. Furthermore, we recorded data about therapeutic management, such as the administration of corticosteroids, antibiotic treatment, inhaled medications, the need and type of oxygen therapy, and the need for endotracheal intubation.

All the clinical information was obtained with full respect for the children’s and their families’ privacy. In accordance with European regulations, Italian observational studies based on data obtained without any additional therapy or monitoring procedures do not need the approval of an independent ethical committee.

The statistical analysis was performed using IBM SPSS Statistics 26.0 (IBM Corp. Armonk, NY, USA). In the first part of the study, we conducted a descriptive analysis: categorical variables were reported as absolute numbers and percentages, and continuous variables were reported as mean and standard deviation (SD), as appropriate. In the last part of the analysis, comparisons between different age groups and between the 2 years of the study were performed. A Student’s *t*-test was used to compare the continuous variables of the study groups. Pearson and Fisher’s exact tests were performed to evaluate the discrete variables, as appropriate. Significance was set at a *p*-value of < 0.05.

## 3. Results

### 3.1. Study Population and Clinical Features

During the study period, 410 hospitalisation episodes for lower airway diseases were registered, equal to 36.8% of total hospitalisations in the emergency pediatric ward during the analysis period. The mean patient’s age was 2.7 ± 3.21 years, and, in 55.1% of the hospitalisations, the patient was male. In 170 hospitalisation episodes, the diagnosis was bronchitis (41.5%).

All the demographic and anamnestic characteristics of the study population are presented in Table 1. Table 2 summarises diagnostic data, such as radiological findings and laboratory values

Data about treatments are reported in Table 3. We found statistically significant differences (*p* < 0.001) in the use of antibiotics in different diseases: they were administered in bacterial pneumonia (56/56; 100%) but also in viral pneumonia (24/27; 88.9%) and bronchitis (45/170; 26.5%).

### 3.2. Outcomes

The mean length of hospitalisation was 6.71 ± 5.4 days. No death has been recorded. In 36 cases (8.8%), the patient was admitted to the Pediatric Intensive Care Unit, and in 4 cases (1%), the patient underwent invasive procedures.

### 3.3. Comparison Between 2 Years

We compared two years: from 1 June 2021 to 31 May 2022 (2021–2022) and from 1 June 2022 to 30 June 2023 (2022–2023).

In the year 2021–2022, we recorded 208 hospitalisations. The mean age was 2.6 ± 3.4 years, and the median length of the hospitalisation was 7.1 ± 6.4 days. In the year 2022–2023, we recorded 202 hospitalisations. The median age was 2.8 ± 3.0 years, and the median length of the hospitalisation was 6.3 ± 4.2 days.

We compared the reasons for hospitalisation, etiologies, presence of respiratory failure, and the need for oxygen therapy (Table 4).

In 2021–2022, more patients were hospitalised for bronchiolitis, while in 2022–2023, more were hospitalised for bacterial pneumonia. Statistically significant differences between the two years were found in the reason for hospitalisation, with the need for intravenous therapy being more prevalent in 2022–2023. Among different etiologies, in 2021–2022, RSV infection was more frequent, with a statistically significant difference, while in 2022–2023, influenza virus was more frequent. No statistically significant differences were found between other pathogens. More cases of patients with respiratory failure in need of low-flow oxygen therapy occurred in the year 2021–2022 than in 2022–2023. No statistically significant differences were found between admission to the PICU and the need for mechanical ventilation.

### 3.4. Comparison Between Different Age Groups

We also made a comparison between three age groups (Table 5): children aged 1–5 years accounted for 46.8% (n = 192) of the hospitalisations, followed by those aged <1 year (*n* = 151, 36.8%) and >5 years (*n* = 67, 16.3%).

The first diagnosis of discharge was bronchiolitis in the age group <1 year (96 cases) and bronchitis in the age group 1–5 years (104 cases) and >5 years (26 cases).

Statistically significant differences between the age groups were found regarding the reason for hospitalisation: patients <1 year were more likely to be hospitalised for respiratory failure, while the need for intravenous therapy was the prevalent reason for the hospitalisation of children aged 1–5 years and >5 years. Among the presentation symptoms, respiratory distress and hypoxia were more frequent in patients <1 year, while fever and cough were more frequent in patients aged 1–5 years. In patients <1 year, RSV was more often positive with a statistically significant difference. No statistically significant difference was found for other pathogens. Respiratory failure and the use of HFNC were more frequent in the age groups <1 year and 1–5 years, with a statistically significant difference between age groups. Regarding therapies, statistically significant differences were found in aerosol therapy, which was more frequent in patients <1 year. Antibiotic therapies were prevalent in children aged 1–5 years, with no differences between beta-lactams and macrolides.

## 4. Discussion

In this retrospective study, we analysed the characteristics of patients admitted to our hospital’s emergency paediatric ward with a diagnosis of lower airway disease.

Multiple studies focus on hospitalisations for lower respiratory tract infections with a particular aetiology or asthma [9,10,11,12]. Still, the international literature lacks epidemiological studies on all the lower airway diseases in a general paediatric population.

In our study, these pathologies account for more than one-third (36.8%) of the overall hospitalisations in the emergency paediatric ward during the analysis period. This observation confirms that paediatric respiratory emergencies are among the most common reasons for emergency department visits and subsequent hospital admissions [13,14,15].

Consistent with previous studies regarding lower respiratory tract infections [9,12], our population showed a slight male-to-female prevalence. The age group 1–5 years was the most represented; lower respiratory infections were more frequent in children <5 years [2].

In the majority of the episodes of hospitalisation, the diagnosis was bronchitis (41.5%). In the literature [16], it is described that more than one-third of infants suffer from wheezing and almost one-fifth from recurrent wheezing, so it is a common pathology, especially in the age group 1–5 years, and frequently, children needing oxygen therapy should be hospitalised. According to the literature [17], and in our study, viral bronchiolitis was the most frequent lower respiratory tract infection and the leading cause of hospitalisation in infants under twelve months of age.

We observed that respiratory failure is the primary reason for hospitalisation (83.9%), with patients <1 year more at risk of respiratory distress and respiratory failure. Factors contributing to rapid respiratory compromise in children include the anatomy of the respiratory tract with smaller airways, increased metabolic demand, decreased respiratory reserve, and inadequate compensatory mechanisms compared with adults [13].

In lower respiratory tract infections, clinical presentation and epidemiologic considerations usually suggest an etiologic agent. According to the British Thoracic Society’s guidelines [18], a microbiologic diagnosis should be established in children with severe disease, potential complications, or those requiring hospitalisation. We observed that frequently, we requested RSV antigenic nasopharyngeal swabs or PCR analysis on the nasopharyngeal swabs to determine the aetiology and to enable correct patient isolation. According to the literature [4,19,20], and also in our study, rhinovirus and RSV were the leading causes of lower respiratory tract infections, and RSV was more common in children <1 year of age. Blood cultures were only performed for patients with severe diseases. Serologies, especially for Mycoplasma and pneumococcal urinary antigen, were performed in a minority of patients, even if the latter should not be performed according to guidelines [18] because of false-positive reactions.

Point-of-care lung ultrasonography (POCUS) is becoming a widely used supplementary tool in evaluating children with acute respiratory distress; it is safe and non-invasive [21]. Although, according to guidelines [18], radiographs should only be requested in children with severe, complicated, or recurrent pneumonia. In our centre, the use of POCUS in the emergency department is still growing; we observed that in 42% of cases, POCUS was performed, but we still require many X-rays. We observed that chest radiography was performed on about half of our patient population. Fortunately, CT scans were performed on a few patients with severe and complicated diseases.

Patients needed oxygen therapy in most episodes, and low-flow nasal cannulae were the first choice [3]. But in nearly one-third of the cases, patients needed high-flow nasal cannulae; HFNC was mainly used in patients <1 year, which is not unexpected because in this age group, bronchiolitis is frequent, and often, the use of HFNC can avoid invasive ventilation [22].

Regarding the therapeutic approach, antibiotics were administered in 45.6% of the episodes, mostly beta-lactams, which are the first choice in cases of bacterial pneumonia [18]. However, we observed that we still prescribe antibiotics for viral diseases (88.9% of viral pneumonia and 26.5% of bronchitis). This result suggests that we can improve antibiotic stewardship.

Regarding outcomes, only 8.8% of the episodes involved admission to the PICU, and 2.9% required mechanical ventilation, in contrast with other studies [9] in which more patients required intensive care, probably due to the smaller population that we analysed.

Comparing the two years of study, in 2021–2022, we recorded more hospitalisations for bronchiolitis, with RSV being the primary pathogen detected. In contrast, influenza activity was minimal, in accordance with the literature [23]. Before the pandemic, RSV and influenza had a clear seasonal pattern, which was interrupted in 2020. The restrictive measures adopted against the SARS-CoV-2 virus drastically reduced respiratory pathogens; in particular, bronchiolitis almost disappeared during the 2020–2021 season [24,25]. With the decrease in social isolation measures, an increase in RSV cases in 2021–2022 exceeding the median seasonal peak was detected, with an earlier peak of shorter duration than pre-pandemic seasons, as reported in other studies [12,23,26,27]. This may be due to the increasing number of children that were naïve to RSV.

No differences were found in the severity of respiratory diseases (considering admissions to PICU and rate of mechanical ventilation) between 2021–2022 and 2022–2023. However, we observed that more patients were admitted to the hospital for respiratory failure and needed low-flow oxygen therapy in 2021–2022 because of the peak in RSV bronchiolitis.

According to the literature [28], low influenza activity was registered in 2021–2022 despite the suspension of strict pandemic control measures. Possible reasons include mask-wearing, social distancing, and a reduction in travel to prevent influenza from spreading. With the elimination of all these restrictive measures, influenza A showed a normal seasonality in 2022–2023.

In contrast with other studies [23], we did not observe differences in the number of diseases caused by other pathogens, probably because of the small population that we analysed.

In 2022–2023, we registered more admissions for bacterial pneumonia and, consequently, an increased need for intravenous therapy. As described in other studies [29,30], this increase could be driven by post-pandemic changes in the pathogenesis of endemic respiratory infections and possibly reflects a high circulation of respiratory pathogens in a susceptible population. A resurgence in viral infections predisposing individuals to bacterial pneumonia in 2022–2023 may have influenced the high incidence.

Our analysis’s strengths include the large study population size and the variety of diseases analysed.

Limitations of this study include its retrospective design and the lack of some data, either because they were not detected or simply not recorded in the clinical records. Another limitation is that we considered hospitalisation episodes, and our observation was limited to patients hospitalised in one of our hospital’s departments.

## 5. Conclusions

Lower respiratory tract diseases are frequent in the pediatric population, and children <5 years old are at higher risk of lower respiratory tract infections. The main reason for admission to hospital is respiratory failure, and this risk is higher in infants, so its management should be part of the professional skills of children’s physicians.

All paediatricians should consider implementing bedside lung ultrasound in lower airway infections because it is quickly available and non-invasive. When possible, X-rays should be limited to complicated and severe diseases [31].

The therapeutic approach should be guided by clinical signs, epidemiology, and laboratory/radiological exams, and the use of antibiotics should be limited in viral diseases, which are the most common in the pediatric population.

Finally, by analysing the differences between the two years of study, we have underlined how the COVID-19 pandemic has changed the epidemiology of acute respiratory infections in children.

## Figures and Tables

**Table 1:** Demographic and anamnestic characteristics of the study population.

	N. Hospitalisations (%)
Sex	
Male	226 (55.1)
Female	184 (44.9)
Age	
<1 year	151 (36.8)
1–5 years	192 (46.8)
>5 years	67 (16.3)
Origin	
Europeans	352 (85.9)
Non-Europeans	58 (14.1)
Symptoms at admission	
Cough	326 (79.5)
Respiratory distress	312 (76.1)
Fever	248 (60.5)
Hyporexia	188 (45.9)
Reason for hospitalisation	
Respiratory failure	344 (83.9)
Need for intravenous therapy	44 (10.7)
Dehydration	13 (3.2)
Other	9 (2.2)
Diagnosis	
Bronchitis	170 (41.5)
Bronchiolitis	99 (24.1)
Bacterial pneumonia	56 (13.7)
Bronchopneumonia	27 (6.6)
Viral pneumonia	27 (6.6)
Asthma	13 (3.2)
Other	18 (4.4)

**Table 2:** Diagnostic data.

	N. Hospitalisations (%)
RSV antigenic nasopharyngeal swab	125 (30.5)
RSV positive	87 (69.6) *
PCR analysis on nasopharyngeal swab	168 (41)
Nasopharyngeal swab positive	139 (82.7) *
Rhino/Enterovirus	42 (25) *
Human metapneumovirus	15 (8.9) *
Adenovirus	8 (4.8) *
Parainfluenza virus	19 (11.3) *
Coronaviruses	8 (4.8) *
Influenza A virus	26 (15.5) *
Influenza B virus	4 (2.4) *
RSV	41 (24.4) *
Serology testing	29 (7.1)
Bacterial cultures	76 (18.5)
Pneumococcal urinary antigen	41 (10)
Pneumococcal urinary antigen-positive	15 (36.6) *
Point-of-care lung ultrasonography	173 (42.2)
Pathological findings	147 (85) *
Chest radiography	201 (49)
Pathological findings	168 (83.6) *
Chest computed tomography (CT)	14 (3.4)
Pathological findings	14 (100) *

* percentage includes only the subjects who performed the exam.

**Table 3:** Therapies administered.

	N. Hospitalisations (%)
Oxygen therapy	338 (82.4)
Low-flow nasal cannulae/mask	257 (62.7)
High flow nasal cannulae (HFNC)/Venturi mask	128 (31.2)
Mechanical ventilation	12 (2.9)
Aerosol therapy	358 (87.3)
Nebulised adrenaline	102 (24.9)
Nebulised bronchodilators	288 (70.2)
Nebulised steroids	95 (23.2)
Oral corticosteroids	242 (59)
Intravenous corticosteroids	73 (17.8)
Intravenous magnesium sulphate/aminophylline/salbutamol	38 (9.3)
Antibiotic treatment	187 (45.6)
Beta-lactam antibiotics	164 (40)
Macrolides antibiotics	50 (12.2)

**Table 4:** Statistically significant differences between the two years of study.

	Year 2021–2022 N. Hospitalisations	Year 2022–2023 N. Hospitalisations	*p*-Value *
Diagnosis			0.026
Asthma	7	6	
Bronchiolitis	**62**	37	
Bronchitis	82	88	
Bronchopneumonia	15	12	
Viral pneumonia	16	11	
Bacterial pneumonia	20	**36**	
Other	6	12	
Reason for hospitalisation			0.016
Respiratory failure	186	158	
Need for intravenous therapy	13	**31**	
Dehydration	5	8	
Other	4	5	
RSV positive ^1^	**57**	30	0.001
Influenza A positive ^2^	5	**21**	0.001
Respiratory failure	**196**	170	0.001
Low-flow oxygen therapy	**143**	114	0.026

The bold formatting highlights statistically significant values. * Chi square test was performed to compare categorical variables. ^1^ Patients tested for RSV: year 21–22: 69 year 22–23: 56. ^2^ Patients tested for influenza A: year 21–22: 76 year 22–23: 92.

**Table 5:** Statistically significant differences between the three age groups.

	<1 Year N. Hospitalisations	1–5 Years N. Hospitalisations	>5 Years N. Hospitalisations	*p*-Value
Reason for hospitalisation				<0.001
Respiratory failure	**143**	158	43	
Need for intravenous therapy	3	**25**	**16**	
Dehydration	5	6	2	
Other	0	3	6	
Cough	**132**	146	47	0.003
Fever	70	**143**	34	<0.001
Respiratory distress	**133**	132	45	<0.001
Hyporexia	**96**	76	15	<0.001
RSV positive	**60**	26	1	<0.001
High-flow nasal cannulae (HFNC)/Venturi mask	**63**	47	16	0.001
Aerosol therapy	**142**	162	52	0.001
Antibiotic treatment	41	**105**	**41**	<0.001
Beta-lactam antibiotics	33	95	36	<0.001
Macrolides antibiotics	6	30	14	<0.001

The bold formatting highlights statistically significant values.

## Data Availability

The data presented in this study are available on request from the corresponding author due to the ongoing study.
